# Primary failure of eruption: occlusal and dentoalveolar characteristics in mixed and permanent dentition. A study with cone beam computed tomography

**DOI:** 10.4317/jced.59657

**Published:** 2022-07-01

**Authors:** Rocío-Esther Avalos-Hernández, Luis-Ernesto Arriola-Guillén, Aron Aliaga-Del Castillo, Yalil-Augusto Rodríguez-Cárdenas, Gustavo-Armando Ruíz-Mora

**Affiliations:** 1 Division of Oral and Maxillofacial Radiology, School of Dentistry, Universidad Científica del Sur, Lima, Perú; 2 Division of Orthodontics and Division of Oral and Maxillofacial Radiology, School of Dentistry, Universidad Científica del Sur, Lima, Perú; 3 Postdoctoral Fellow. Department of Orthodontics, Bauru Dental School. University of São Paulo, Brazil; 4 Division of Oral and Maxillofacial Radiology, School of Dentistry, Universidad Científica del Sur, Lima, Perú. Associate Professor of the Division of Oral and Maxillofacial Radiology, School of Dentistry, Universidad Nacional de Colombia, Bogotá D.C, Colombia; 5 Division of Orthodontics, Faculty of Dentistry, Universidad Nacional de Colombia, Bogotá D.C, Colombia; and Associate Professor of the Division of Oral and Maxillofacial Radiology, School of Dentistry, Universidad Científica del Sur, Lima, Perú

## Abstract

**Background:**

The main objective of this study was to describe the dentoalveolar and occlusal characteristics of subjects with primary failure of eruption (PFE) in cone beam computed tomography (CBCT) and compare them with a control group without the anomaly.

**Material and Methods:**

This retrospective and comparative study evaluated CBCT images of 80 permanent molars divided into 2 groups of 40 molars each, which had or did not have PFE characteristics. Using CBCT a calibrated orthodontist performed 23 measurements related to the distances of the cusps to the occlusal, palatal and mandibular planes and measured the buccal-palatal and mesiodistal widths of the molar crowns and their root lengths. Independent Student’s t or Mann-Whitney U tests were used to compare occlusal and dentoalevolar characteristics between groups according to the normality of the data. *P*<0.05.

**Results:**

Molars affected by PFE were smaller in coronal dimensions in the PFE compared to the non-PFE group (Buccal-palatal crown width 11.60±0.95mm vs. 12.21±1.09 mm; *p*<0.001; and mesiodistal crown width 10.81± 1.07 mm vs. 11.84±1.32mm, respectively; *p*<0.001). The distal and mesial root lengths were approximately 2 mm smaller in the PFE group (*p*<0 .001). The three-dimensional bony position was more convergent with respect to the palatal plane, i.e., the crown was more inclined palatally and distally in the PFE compared to the non-PFE group.

**Conclusions:**

Patients with molars affected by PFE present smaller bucco-palatal, mesio-distal coronal widths and mesial and distal root lengths than those without PFE and in these teeth the crowns are more inclined palatally and distally in upper molars. These specific characteristics may be pathognomonic of PFE and should be considered by clinicians when evaluating or treating patients with this condition.

** Key words:**Occlusal characteristics, primary failure of eruption, cone-beam CT.

## Introduction

Primary failure of eruption (PFE) was described by Proffit and Vig (1981) as the malfunction of dental eruptive mechanisms with delay and absence of these processes in a non-ankylosed tooth (1). Under occlusion of the affected tooth, vertical alveolar atrophy, posterior open bites, impaction of neighboring posterior teeth and sometimes anterior teeth, as well as agenesis and/or ankylosis of deciduous molars are the most common characteristics of PFE (2). PFE is preferably unilateral, rarely symmetrical, but can affect any or all of the posterior quadrants (2). PFE and ankylosis are considered to be closely related and have frequently been associated with skeletal class III malocclusions (3).

PFE is an inherited disorder that may affect approximately 0.6% of the population (4-6). Mutations in parathyroid hormone receptor 1 (PTH1R) have been identified and confirmed to be responsible for this eruptive phenotypic expression (4-6). These phenotypes can be found in the deciduous dentition as well as permanent dentition (7-9).

The severity of PFE is variable and therefore treatment should be individualized depending generally on the age of the patient (10). It has been observed that the affected PFE tooth does not respond to orthodontic forces and that a non-ankylosed PFE tooth can become an ankylosed tooth when forces are applied (3,11). In some cases, it is difficult to distinguish PFE from other eruption disorders such as ankylosis and this is key for developing an adequate treatment plan (2). In recent years, different types of treatment have been proposed for patients with this condition, ranging from dental restorations to surgical procedures such as osteotomies (12,13).

The combination of a good clinical diagnosis and genetic analysis can provide a guideline to improve the management of these cases, help in treatment planning to avoid unnecessary therapies for the patient, as well as to detect other relatives potentially affected by PFE (4).

Cone beam computer tomography (CBCT) is a diagnostic tool that allows a three-dimensional (3D) analysis of different eruptive alterations in the jaw (12). On reviewing the scientific literature to date, little is known about 3D dentoalveolar and occlusal characteristics of PFE according to CBCT. For this reason, the purpose of this study was to compare the dentoalveolar and occlusal characteristics obtained by CBCT study in subjects with PFE with those of a control group without the anomaly.

## Material and Methods

This retrospective comparative study was approved by the Research Ethics Committee of the Universidad Científica del Sur, Lima, Peru (registration number 247-2021-POS8). Written informed consent was obtained from all participants and the parents of minors, and all data were fully anonymized prior to access by the investigators. For this study, CBCT images of 80 molars divided into 2 groups were evaluated: Group 1 was composed of 40 molars from 20 patients (8 females and 12 males aged 7 to 20 years) who presented PFE characteristics; and Group 2, or the control group, was composed of CBCT images of 40 molars belonging to 7 patients (3 females and 4 males aged 7 to 20 years) who did not present characteristics related to any eruptive anomaly. This sample selection and distribution was performed by 2 calibrated orthodontists with 10 years of experience. In case of discrepancy, the final concept was defined by consensus.

The sample size for the study group was obtained using a formula to compare two means (root length of teeth with and without PFE), with the following parameters, 95% confidence interval, test power 80%, mean difference of 1.87 mm and ± of 2.11 (data obtained from a previous pilot test). The required sample size was 22 teeth for each group; however, 40 teeth with and without PFE per group were obtained and the sample was similar to previous studies. (14,15) Furthermore, since the prevalence of this condition is low, it was decided to obtain a multicenter sample and the CBCT images were obtained from imaging centers in Honduras, Colombia, Dominican Republic, Peru and Mexico. The characteristics of the sample are described in Table 1 and  Table 2.

**Table 1:**
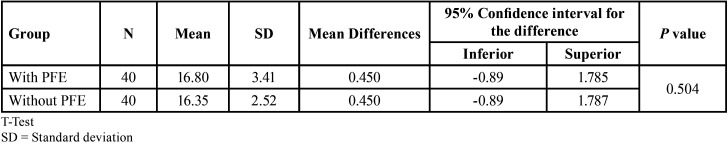
Initial characteristics of the sample.

**Table 2:**
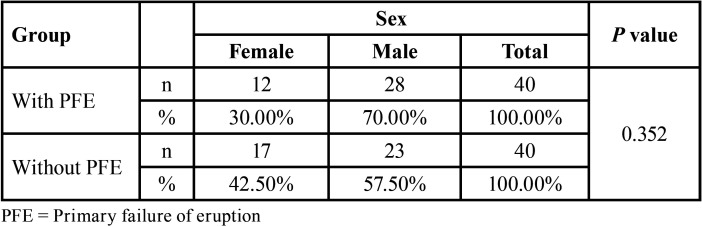
Distribution of the sex of the cases evaluated by group.

The inclusion criteria consisted of: complete unimaxillary or bimaxillary CBCT of healthy, asymptomatic subjects of both sexes; no systemic disease; authorization and consent for the use of images in research; in permanent dentition; older than 12 years; under occlusion of at least one permanent molar or premolar; arrested intraosseous or supraosseous evolution associated with posterior open bite; absence of signs of eruptive potential; and having exceeded the average eruptive period of the population and teeth without signs of ankylosis. Exclusion criteria were: CBCT of syndromic individuals, or total or partial edentulism, with the presence of congenital and dental anomalies, obstruction of the eruptive pathway by the third molar, teeth with a history of dentoalveolar trauma, or CBCT of poor quality.

**Figure 1:**
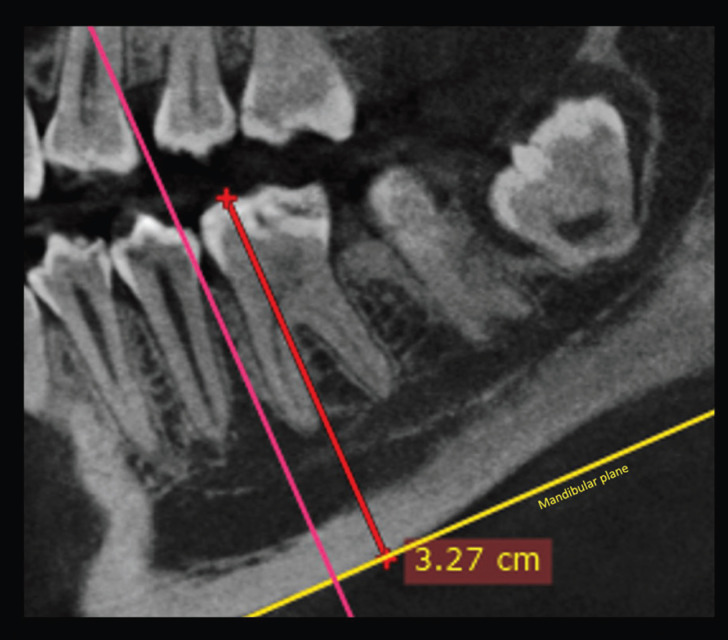
Evaluation of the distance from the mesial cusp to the mandibular plane.

**Figure 2:**
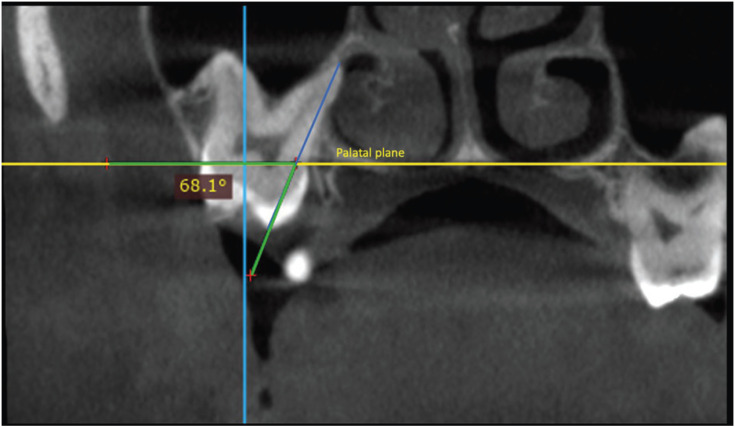
Evaluation of the angle formed between the longitudinal axis with the palatal plane in coronal section (palatal root).

**Figure 3:**
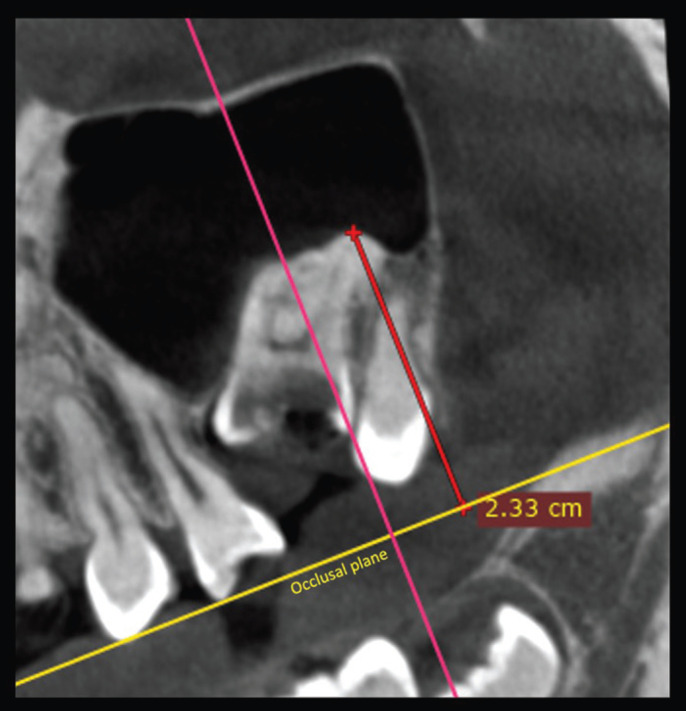
Distance in mm from the distal root to the occlusal plane.

DICOM files were analyzed with Dolphin Imaging software version 11.9 (Dolphin Imaging and Management Solutions, Chatsworth, California 2017). 3D evaluation and description of the dentoalveolar and occlusal characteristics of patients with PFE in the upper and lower jaw were measured using 3D and multiplanar reconstructions. For the measurements of each tomography, the axial and sagittal planes were aligned with the anterior (ANS) and posterior (PNS) nasal spine. In this way, the palatal plane and the mid-sagittal plane were constructed. The occlusal plane was constructed by means of a line drawn along the occlusal edges of the posterior teeth on the volumetric reconstruction. In some cases, only the cusps of the first or second molar and the cusp of the canine were taken as reference. The lengths and angles evaluated are described in Table 3, (Figs. 1-3). A total of 23 measurements of paired teeth were taken in the PFE and non-PFE groups.

**Table 3:**
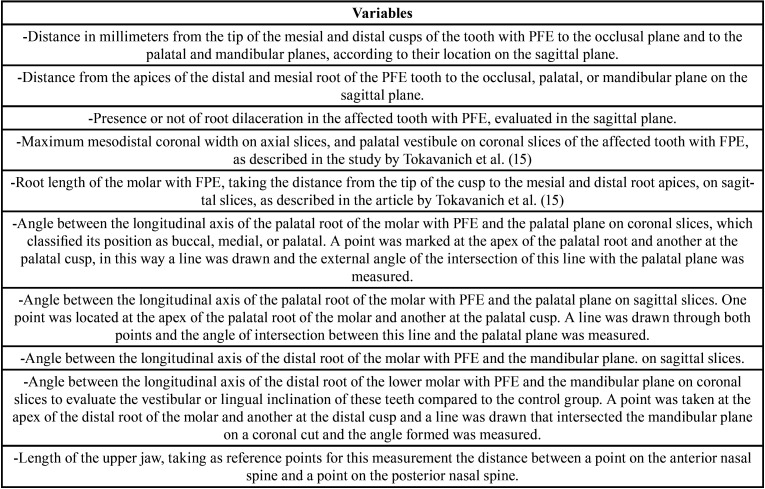
Description of evaluated variables.

All the measurements obtained from the CBCT images were performed by two calibrated dentists with 10 years of experience (R.E.A.H and G.A.R.M). Intra- and interobserver agreement was evaluated with the Kappa coefficient and values greater than 0.85 were obtained. To assess intraobserver reliability with respect to the measurements of change in height, width and depths the same examiner (R.E.A.H.) re-evaluated the sample after a period of 30 days. The data were analyzed with the intraclass correlation coefficient (ICC) which yielded results greater than 0.85 with a confidence interval of 95%. Additionally, the random error was calculated with Dahlberg’s formula, yielding values lower than l mm or 1° for all quantitative variables.

-Statistical analysis

Statistical analysis was performed using SPSS Statistics for Windows (version 21.0; IBM Corp, Armonk, NY). Descriptive statistics were calculated for changes in height, width, and depth in millimeters for both groups. Normality of the data was assessed with the Shapiro-Wilk test. According to the normality of the data, the independent t-test or Mann-Whitney U-test was applied for the comparison of heights, widths and depths between both groups. The level of significance was set at *p*<0.05. Finally, a binary logistic regression was performed to estimate the influence of the predictor variables on the PFE variable.

## Results

The comparisons of the variables evaluated are summarized in Table 4,  Table 4 cont. On comparing the non-PFE with the PFE group, the distance from the distal cusp to the occlusal plane was 0.12 ± 0.55 mm vs. 7.84 ± 4.40 mm, respectively (*p*<0 .001), while the distance from the mesial cusp to the occlusal plane was 0.34 ± 0.64 mm vs. 7.99 ± 3.76 mm in the PFE group, respectively (*p*<0 .001). The distance from the distal cusp to the palatal plane was 20.66 ± 3.32 mm vs. and 9.58 ± 4.86 mm, respectively (*p*<0.001) and the distance from the mesial cusp to the palatal plane was 21.35 ± 2.96 mm vs. 10.86 ± 4.90 mm, respectively (*p*<0.001). Regarding the mandibular plane the distal cusp distance was 30.95 ± of 4.15mm vs. 23.18 ± 2.78 mm, respectively (*p*<0 .001) and the distance from the mesial cusp to the mandibular plane was 32.21± 4.45 mm vs. 23.57 ± 3.38 mm, respectively (PFE group) (*p*<0 .001). The distances of the distal and mesial root to the occlusal plane, palatal plane and mandibular plane also presented differences between the two groups (*p*<0.001). Likewise, the Buccal-palatal crown width was smaller in the non-PFE group compared to the PFE group with 12.21± 1.09mm vs. 11.60 ± 0.95 mm, respectively (*P*<0.001) and the mesiodistal crown width was 11.84 ± 1.32 mm vs. 10.81± of 1.07 mm, respectively (*p*<0.001). Moreover, the distal and mesial root lengths were approximately 2 mm smaller in the PFE group than the non-PFE group (*p*<0.001).

**Table 4:**
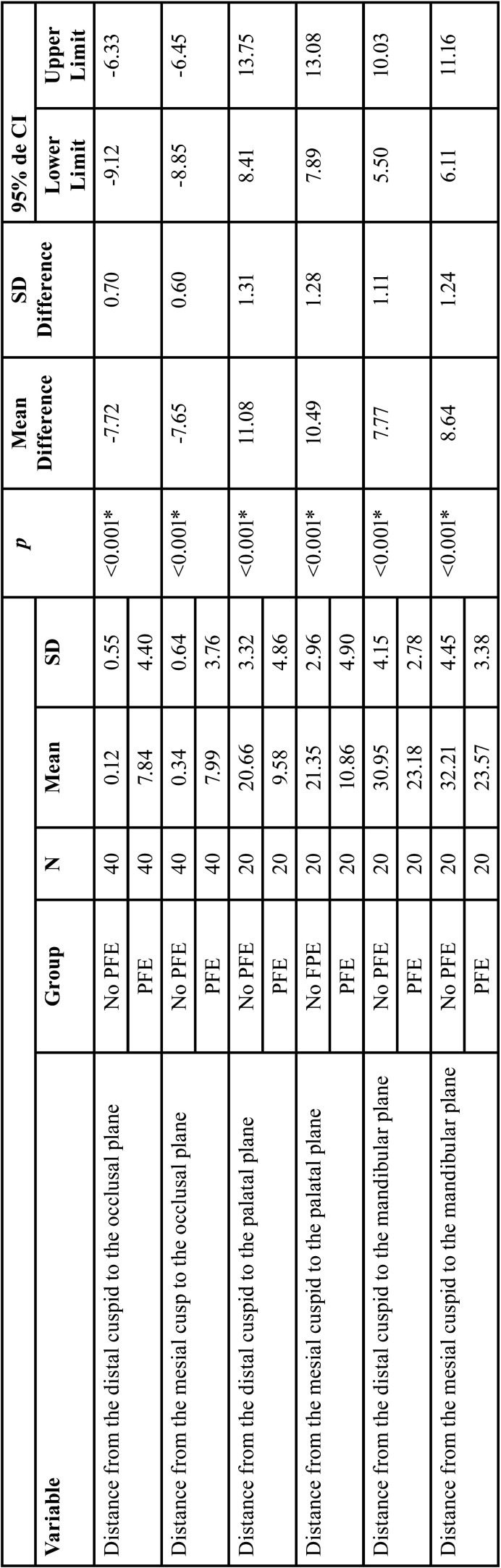
Comparison of the sample according to the presence of PFE: quantitative variables.

**Table 4 cont.**
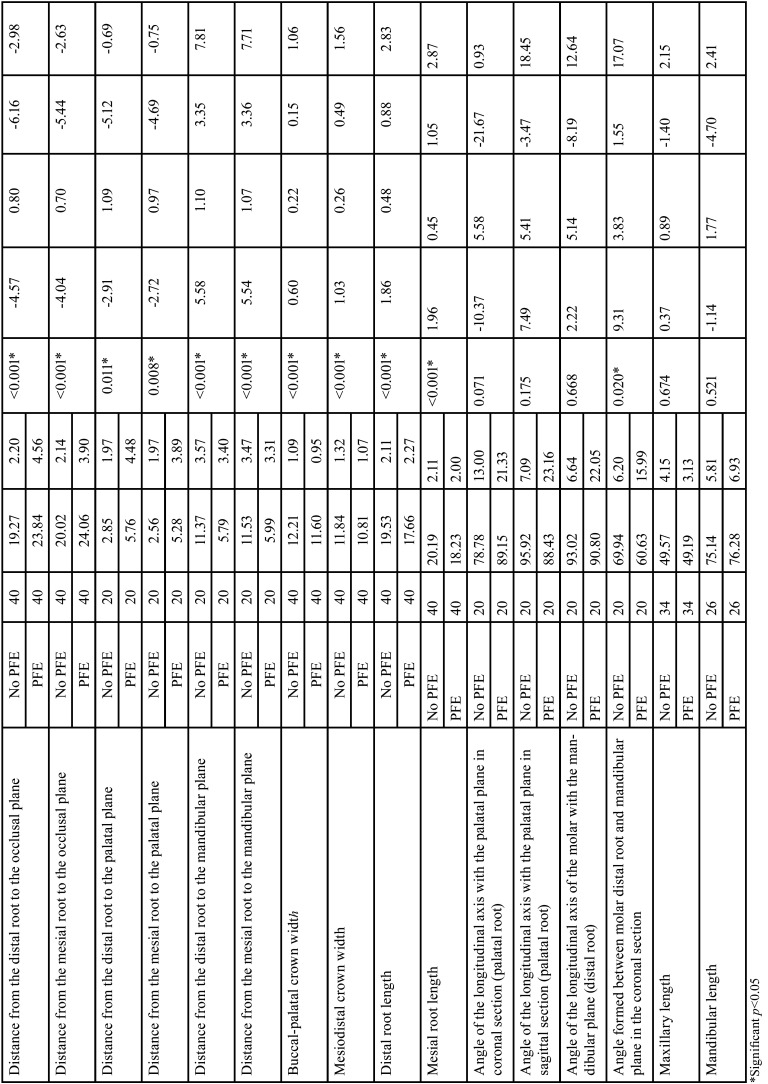
Comparison of the sample according to the presence of PFE: quantitative variables.

On the other hand, significant association was found between root dilaceration and the absence of PFE (62.50%) versus the presence of roots without dilaceration and the presence of PFE (79.20%) (*P*<0.001). These findings are summarized in Table 5. Finally, the binary logistic regression analysis to detect the occurrence of PFE according to the study covariates showed that the presence of root dilaceration could be a protective factor for presenting PFE (*p*=0.016) (Table 6).

**Table 5:**
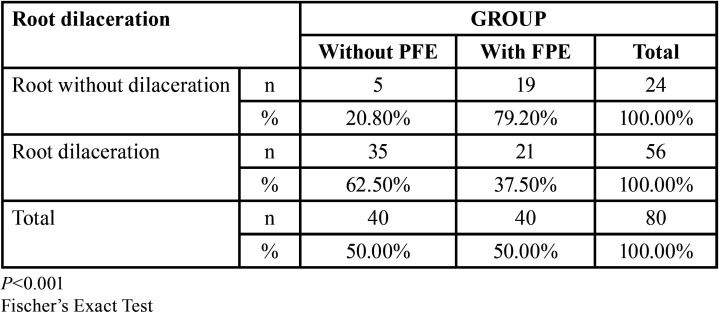
Association between root dilaceration and PFE.

**Table 6:**
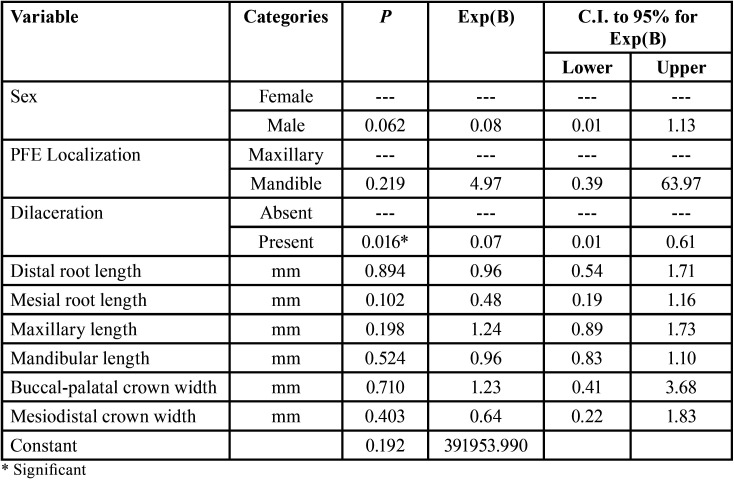
Binary logistical regression analysis to evaluate the influence of all predictor variables on PFE.

## Discussion

PFE is a disorder that has also been investigated under a genetic approach. In the present study, we evaluated some of the dentoalveolar and occlusal characteristics of a group of 40 teeth with PFE and compared these characteristics with a group of 40 teeth without PFE by CBCT imaging.

Compared to normal teeth, teeth affected by PFE present special characteristics. While the significant differences found between groups in the distances to the occlusal plane and palatal or mandibular plane were predictable, the remaining measurements make the findings of the present study of clinical and imaging significant. Using the mesial and distal roots as references, the coronal dimensions (bucco-palatal and meso-distal width) and root length were smaller in the molars affected by PFE. Regarding their 3D osseous position, they were more convergent with respect to the palatal plane, that is, the crowns were more inclined towards the palatine and distal than those without PFE. However, this crown behavior is apparently exclusive of maxillary PFE molars, because it was not found in mandibular molars. This was confirmed by the angle formed between the axis and the mandibular plane, in which no significant differences were found with the control group. All the above characteristics could be defined as pathognomonic of PFE.

These differences are important to achieve early diagnosis, and especially in the development of a differential diagnosis with ankylosed teeth. The differentiation between these two entities is difficult, but our imaging findings can shed light on correct identification. Although not comparable, these findings are consistent with the morphological abnormalities of the root and crown of molars with PFE observed in studies in mice by Tokavanich *et al*. (16), Cohen-Lévy and Cohen (17), Deffrennes and Cohen-Lévy (10), Pilz *et al*. (18), in which PFE was associated with tooth root anomalies and normal crown morphology. Likewise, Vedtofte *et al*. (19) reported that arrested eruption of molars was related to root and crown morphological abnormalities of the affected molar.

With regard to the inclination of molars with PFE, this study was based on angles similar to those obtained by Tong *et al*. (20) who proposed a method to determine the vestibulolingual inclination of molars from tomographic images taking the occlusal plane as a reference point. The findings of Grosso *et al*. (21) showed that the inclination of first molars is on average 88.49 ± 5.39 ° and 84.78 ± 5.99 ° in second molars, similar to the results of the present study. Additionally, Eraydin *et al*. (22) reported a molar inclination in the vestibulo-palatal direction of 81.8 ± 5.0, findings also coinciding with those of the present study, and indicating that upper molars with PFE have a tendency to present palatal inclination. As for the lower molars, Eraydin *et al*. (22) reported an average bucco-lingual inclination of the first molar of 105.7 ± 7.0 and of 108.9 ± 7.0 of the second lower molar, which according to our results would indicate that lower molars with PFE have a tendency to present a vestibular inclination.

We found significant association regarding root dilaceration and the absence of PFE, although this result is different from that reported in the literature, our results found an association between root dilaceration and the absence of PFE different to what was described by Grippaudo *et al*. (14) who stated that root dilacerations in molars with PFE are very rare and are not discriminative for PFE-related PTH1R mutations. Pilz *et al*. (18) also stated that root dilaceration is not a parameter to consider for the differential diagnosis of PFE, and Palma *et al*. (23) reported that there was no statistically significant association between PFE and root dilaceration. While the study by Tokavanich *et al*. (16) is not comparable with the present study due to the type of sample, these authors did associate root dilaceration with PFE in the mouse molars evaluated.

Moreover, the binary logistic regression performed to detect the occurrence of a PFE according to the study covariables indicated that the only protective covariate for PFE is root dilaceration. Our results regarding the risk factors for presenting PFE indicate that teeth presenting root dilaceration have a probability of approximately 62.50% of not presenting PFE. On the other hand, teeth without root dilaceration would have an approximate 79.20% probability of presenting PFE. In this way, our results do not coincide with Palma *et al*. (23) who reported that the presence of root dilaceration determines a poor prognosis. On the other hand, Cohen-Lévy *et al*. (17) and Standerwick (24) stated that root dilaceration is a consequence of decreased tooth movement. However, our study as well as others by Grippaudo *et al*. (14) and *Pi*lz *et al*. (18) show that the percentage of molars with PFE without root dilaceration was higher than that of molars with PFE with root dilaceration. Therefore, our findings could contribute to clarifying the imaging diagnosis of PFE.

Finally, this article concluded that the molars affected with PFE present smaller coronal dimensions (bucco-palatal and meso-distal width) and mesial and distal root lengths than those without PFE. Likewise, the crowns of teeth with PFE are more palatally and distally inclined in upper molars but this is not observed in lower molars. These features may be pathognomonic of PFE. Nonetheless, our study has some limitations such as the heterogeneity of the study group sample, which was composed of maxillary and mandibular first and second molars with PFE. Thus, the sample for each subdivision and location of PFE was small. More studies are needed to further clarify and elucidate these results.

## Conclusions

Molars affected by PFE have smaller coronal dimensions (bucco-palatal and meso-distal width) and smaller mesial and distal root lengths than those without PFE. Likewise, in these teeth the crowns are more inclined palatally and distally in upper molars. These specific features may be pathognomonic of PFE and should be considered by clinicians when evaluating or treating patients with possible PFE.
